# Nitrous Oxide as a Recreational Drug of Abuse: Multi-Omics Insights into MAPK/ERK/CREB-Mediated Neurotoxicity and Energy Metabolism Collapse

**DOI:** 10.3390/molecules31162844

**Published:** 2026-08-14

**Authors:** Juan Jia, Wen Zhang, Sitong Nan, Haiyun Liu, Qian Xue, Congying Liu, Keming Yun, Jiangwei Yan

**Affiliations:** 1 School of Forensic Medicine, Shanxi Medical University, Jinzhong 030600, China; jiajuan@sxmu.edu.cn (J.J.); 13920527687@163.com (W.Z.); 18835978077@163.com (S.N.); 19847525380@163.com (H.L.); 18384561167@163.com (Q.X.); 15757974250@163.com (C.L.); yunkeming5142@163.com (K.Y.); 2 Shanxi Key Laboratory of Forensic Medicine, Jinzhong 030600, China; 3 Shanxi Province Engineering Research Center of Forensic Identification, Jinzhong 030600, China; 4 Key Laboratory of Forensic Toxicology of Ministry of Public Security, Jinzhong 030600, China

**Keywords:** nitrous oxide, recreational drug abuse, neurotoxicity, MAPK/ERK/CREB pathway, energy metabolism collapse, multi-omics

## Abstract

Nitrous oxide (N_2_O), commonly known as laughing gas, is widely used for its anesthetic and analgesic properties in medical settings. However, its recreational abuse has escalated, leading to severe neurological complications including cognitive impairment, yet the underlying mechanisms remain poorly understood. In this study, male *C57BL/6 J* mice were exposed to either normal air inhalation or nitrous oxide inhalation for 28 days to evaluate the effects of repeated nitrous oxide exposure on cognitive function. We employed hippocampal transcriptome sequencing, real-time PCR, Western blotting, and non-targeted plasma metabolomics to explore potential regulatory mechanisms. Repeated nitrous oxide exposure impaired motor skills, learning, and cognitive abilities in mice. Integrative multi-omics analysis revealed that differentially expressed metabolites and genes synergistically disrupted energy metabolism through co-regulation of the TCA cycle, MAPK signaling pathway, and pyrimidine metabolism, ultimately leading to impaired cellular signaling, nucleic acid synthesis, and cognitive dysfunction. Our study provides novel multi-omics insights into the MAPK/ERK/CREB-mediated neurotoxicity and energy metabolism collapse induced by nitrous oxide as a recreational drug of abuse, offering new directions for clinical intervention.

## 1. Introduction

Nitrous oxide is commonly known as “laughing gas” due to the brief, rapid onset of euphoria and dissociative effects it produces upon inhalation, often accompanied by spontaneous laughter [1,2]. In medical settings, nitrous oxide serves as an inhalation anesthetic, commonly used in dental extractions, obstetric labor analgesia, and gastrointestinal endoscopy procedures, offering significant analgesic effects. However, the recreational use has seen an escalating trend in recent years [3]. Long-term excessive inhalation and abuse of nitrous oxide can cause irreversible damage to multiple organ systems, particularly the nervous system [4,5]. While clinical cases have documented nitrous oxide-induced cognitive impairment, the precise mechanisms underlying this effect remain unclear [6,7]. Therefore, it is essential to select appropriate animal models and leverage modern biotechnologies to comprehensively investigate inter-organ injury relationships, identify key target organs, and elucidate toxicity mechanisms for effective preclinical research and clinical practice.

Cognitive impairment refers to pathological processes involving abnormalities in higher-level cognitive processing related to learning, memory, and reasoning, leading to severe learning and memory deficits accompanied by conditions such as aphasia, agnosia, or apraxia [8,9,10]. The causes of cognitive impairment are diverse, with cardiovascular factors and age being significant contributors [11,12]. In recent years, with the increasing severity of nitrous oxide abuse, clinical cases of cognitive impairment caused by nitrous oxide abuse have been frequently reported. Srichawla [13] administered the Mini-Mental State Examination (MMSE) to long-term nitrous oxide users (continuous use for three months, averaging 80–100 canisters daily) and revealed mild cognitive decline with a score of 22. Specific deficits were observed in object recall and calculation. Fan [14] conducted clinical observations and analyses of 63 nitrous oxide abusers. All patients exhibited varying degrees of emotional instability, irritability, anxiety, severe insomnia, and psychiatric symptoms such as auditory and visual hallucinations and delusions. Forty-four patients presented with clinical manifestations including varying degrees of limb muscle weakness or paralysis, inability to walk, and cognitive impairment. Therefore, investigating the mechanisms by which nitrous oxide induces cognitive impairment holds significant value for aiding clinical treatment of cognitive dysfunction.

This study aims to assess the effects of nitrous oxide on the nervous system in mice. To systematically and comprehensively describe the potential mechanisms underlying nitrous oxide-induced cognitive impairment, an integrated transcriptomics and metabolomics analysis was employed. This approach preliminarily identified potential key molecular targets and their associated signaling pathways, providing insights that may inform the clinical management of cognitive impairment associated with nitrous oxide exposure.

## 2. Results

### 2.1. Evaluation of Cognitive Impairment Model

**Figure 1:**
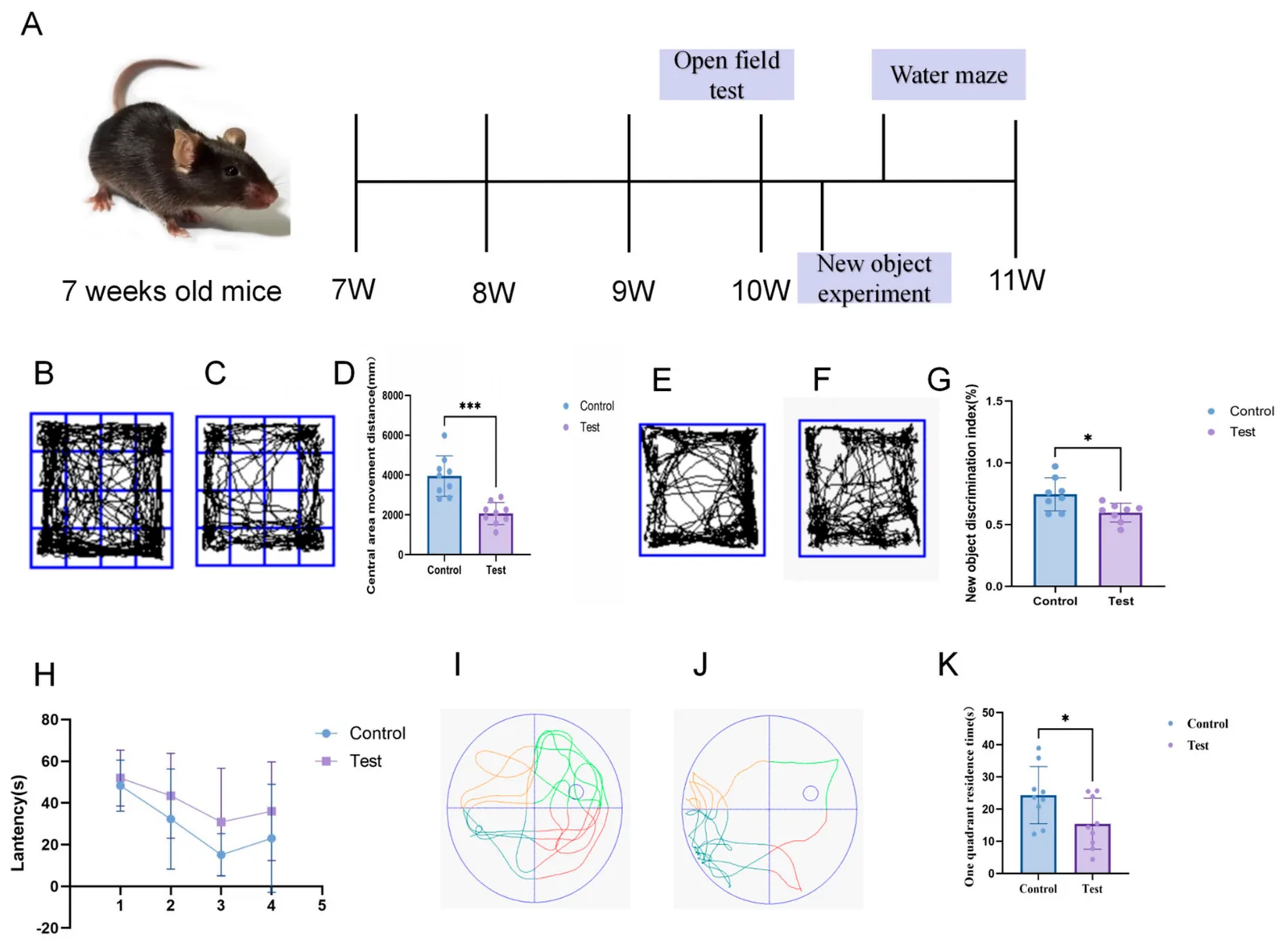
Nitrous oxide induces cognitive impairment in mice (*n* = 9). (**A**): Flowchart of mice behavioral experiment; (**B**,**C**): Mouse open-field trajectory plot ((**B**): control group open-field test plots; (**C**): Experimental group open-field test plots); (**D**): Statistical analysis of central path length in the open field of mice (*** *p* < 0.001; ANOVA and independent-samples Student’s *t*-test); (**E**,**F**): Trajectory diagram of new object recognition test in mice ((**E**): Trajectory diagrams of new object recognition test in the control group; (**F**): Trajectory diagrams of new object recognition test in the experimental group), (**G**): Statistical analysis of novel object recognition index in mice (* *p* < 0.05; ANOVA and independent-samples Student’s *t*-test), (**H**): Statistical analysis of water maze escape latency in mice, (**H**): The effect of nitrous exposure on the localization navigation period in mice. (**I**,**J**): Morris water maze trajectory map in mice ((**I**): Morris water maze trajectories of the control group; (**J**): Morris water maze trajectories of the experimental group), (**K**): Statistical analysis of activity time in one quadrant of water maze in mice (* *p* < 0.05; ANOVA and independent-samples Student’s *t*-test).

To investigate the effects of repeated nitrous oxide exposure on cognitive function and its potential mechanisms, male *C57BL/6J* mice underwent a 4-week nitrous oxide exposure regimen. Behavioral assessments were conducted during this period (Figure 1A). The validity of model was assessed using the open field test, novel object recognition test, and water maze test. Results showed that in the open field test, the experimental group exhibited significantly reduced central activity compared to the control group, indicating increase anxiety-like behavior (*p* < 0.001, Figure 1B–D). In the novel object recognition test, experimental mice spent less time exploring the novel object (*p* < 0.05, Figure 1E–G), indicating impaired recognition memory due to repeated nitrous oxide exposure. The Morris water maze results showed that during the training phase, experimental mice exhibited longer escape latency on days 1–4 compared to control mice (Figure 1H). During the test phase, the experimental group mice exhibited significantly lower quadrant dwell time and fewer platform crossings than the control group (*p* < 0.05, Figure 1I–K), indicating worsened cognitive impairment in the experimental group.

### 2.2. Analysis of Hippocampal Tissue HE Staining

**Figure 2:**
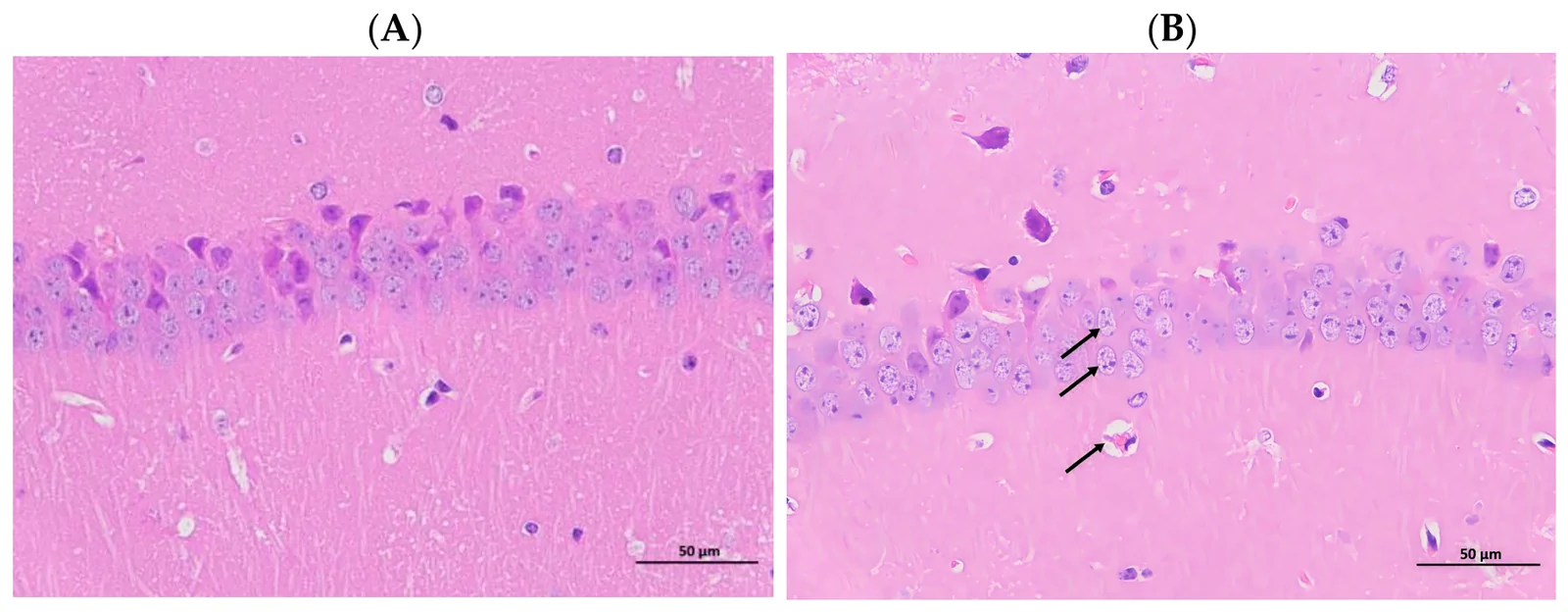
HE staining of hippocampal tissue. (**A**) Blank group; (**B**) Experimental group. (Arrows in (**B**) indicate loosely arranged and lightly stained cells, cellular edema, and scattered satellite cells in the experimental group).

The hippocampus plays a crucial role in the storage of episodic memory and spatial navigation. Damage to the hippocampus may lead to neurodegenerative changes [15]. Therefore, we subsequently investigated the effects of nitrous oxide exposure on hippocampal tissue damage in mice. Hematoxylin and eosin (HE) staining results revealed that, compared with the control group, the experimental group exhibited loosely arranged and lightly stained cells, cellular edema, and scattered satellite cells, indicating neurotoxic changes (Figure 2). These histological alterations may contribute to the development of cognitive impairment in mice.

### 2.3. Analysis of Hippocampal Tissue Immunofluorescence Staining

**Figure 3:**
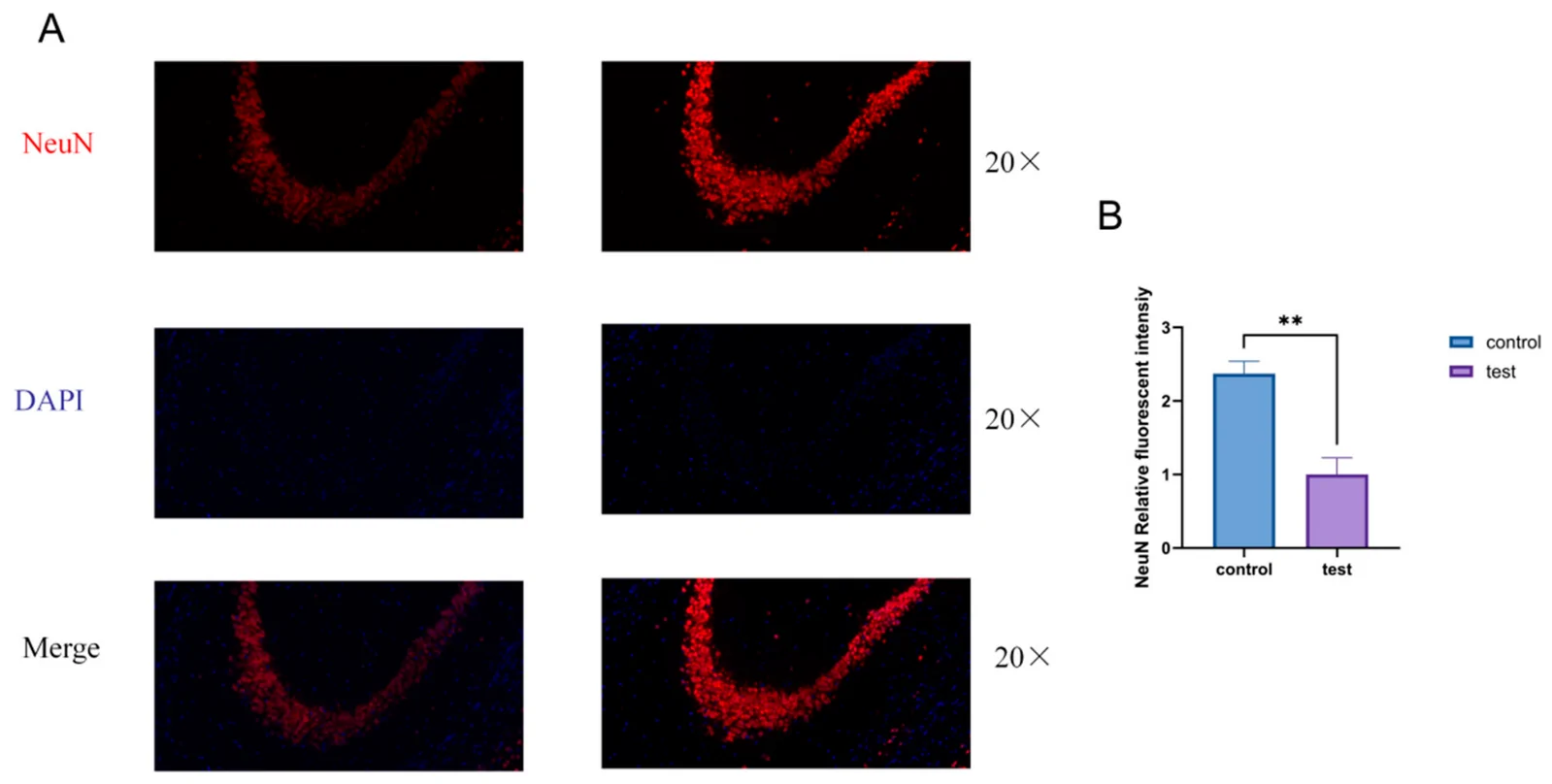
Immunofluorescence Staining of hippocampal tissue (*n* = 3). (**A**) Expression of NeuN was determined by immunofluorescence. (20×). (**B**) The change in the number of NeuN positive cells in hippocampa of each group, ** *p* < 0.01 (ANOVA and independent-samples Student’s *t*-test).

In the experimental group, the number of NeuN-positive neurons in the hippocampal region was significantly reduced, accompanied by disorganized neuronal arrangement (Figure 3), which may constitute the neuropathological basis for impairments in learning and memory abilities.

### 2.4. Transcriptomic Analysis of Hippocampal Tissue

**Figure 4:**
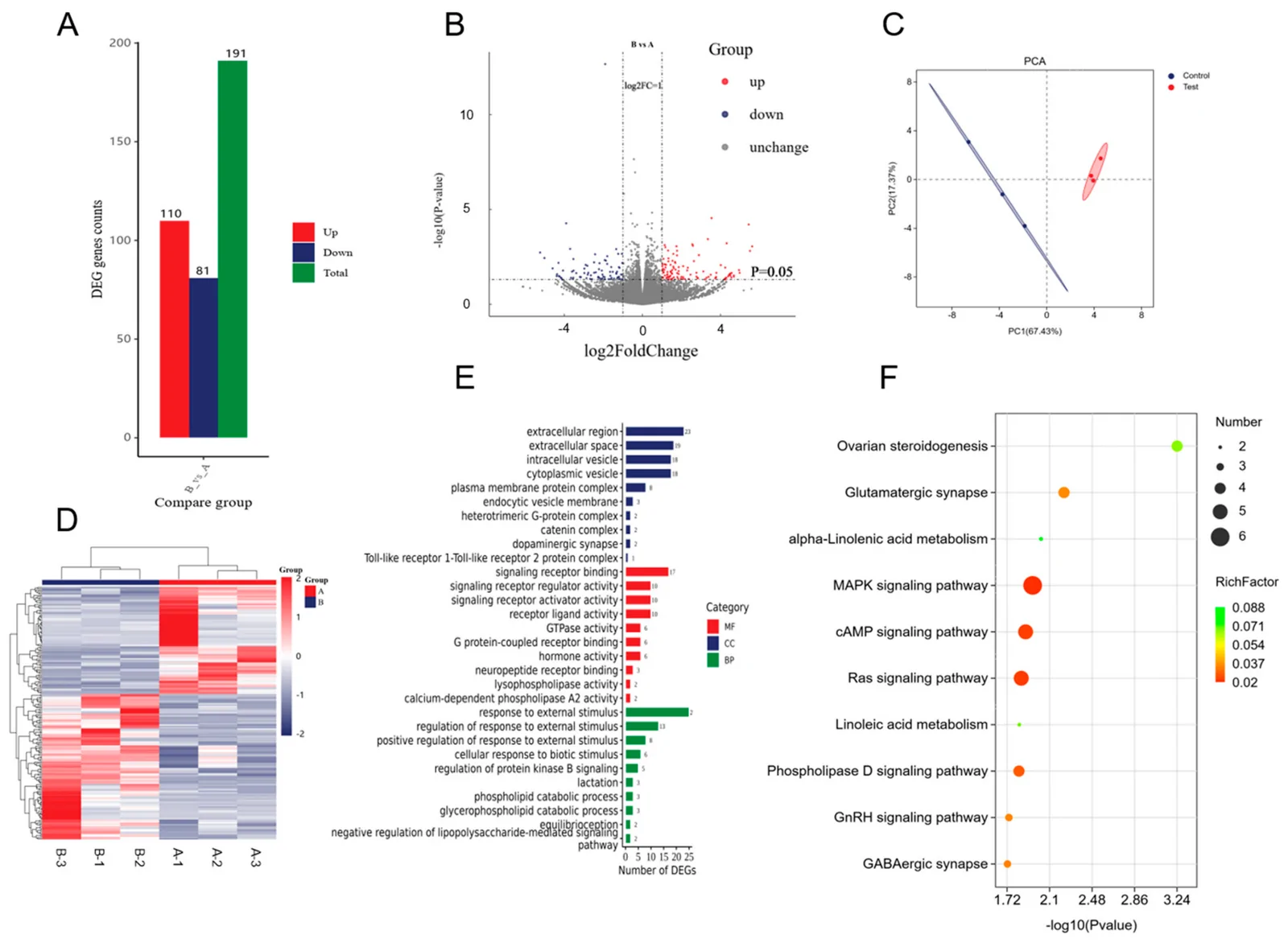
Nitrous oxide alters mRNA expression in the mice hippocampus (*n* = 3). (**A**): Differentially expressed genes bar chart, (**B**): The volcano map of hippocampal differential genes, (**C**): PCA of the overall sample, (**D**): Heat map of differential genes, (**E**): GO functional enrichment analysis diagram of differentially expressed genes, (**F**): Bubble chart of enrichment analysis of differential gene metabolic pathways.

To elucidate how repeated nitrous oxide exposure induces cognitive impairment in mice, we characterized hippocampal gene expression profiles in mice subjected to long-term nitrous oxide administration. Using |log_2_FC| ≥ 1 and *p* < 0.05 as screening criteria, 191 differentially expressed genes (DEGs) were identified (Figure 4A, Table S1). These comprised 110 upregulated DEGs and 81 downregulated DEGs, as shown in the volcano plot (Figure 4B). Principal component analysis (PCA) revealed a clear separation between control and experimental groups (Figure 4C). Differentially expressed genes within each group were further visualized as a clustered heatmap (Figure 4D). Additionally, Gene Ontology (GO) enrichment analysis was performed to identify key biological processes associated with the DEGs. Interestingly, GO enrichment analysis of DEGs identified biological processes such as regulation of response to external stimuli, cellular response to biological stimuli, and protein kinase B signaling. Major changes in DEGs within cellular components were concentrated in the extracellular region, cytoplasmic vesicles, and other regions. Molecular function analysis identified diverse functions for DEGs, including signal receptor regulatory activity, signal receptor activation activity, and hormone activity (Figure 4E). KEGG pathway analysis further revealed that DEGs were predominantly enriched in serotonergic synapse, MAPK signaling pathway, and cAMP signaling pathway (Figure 4F).

### 2.5. Metabolomic Data Analysis of Plasma

**Figure 5:**
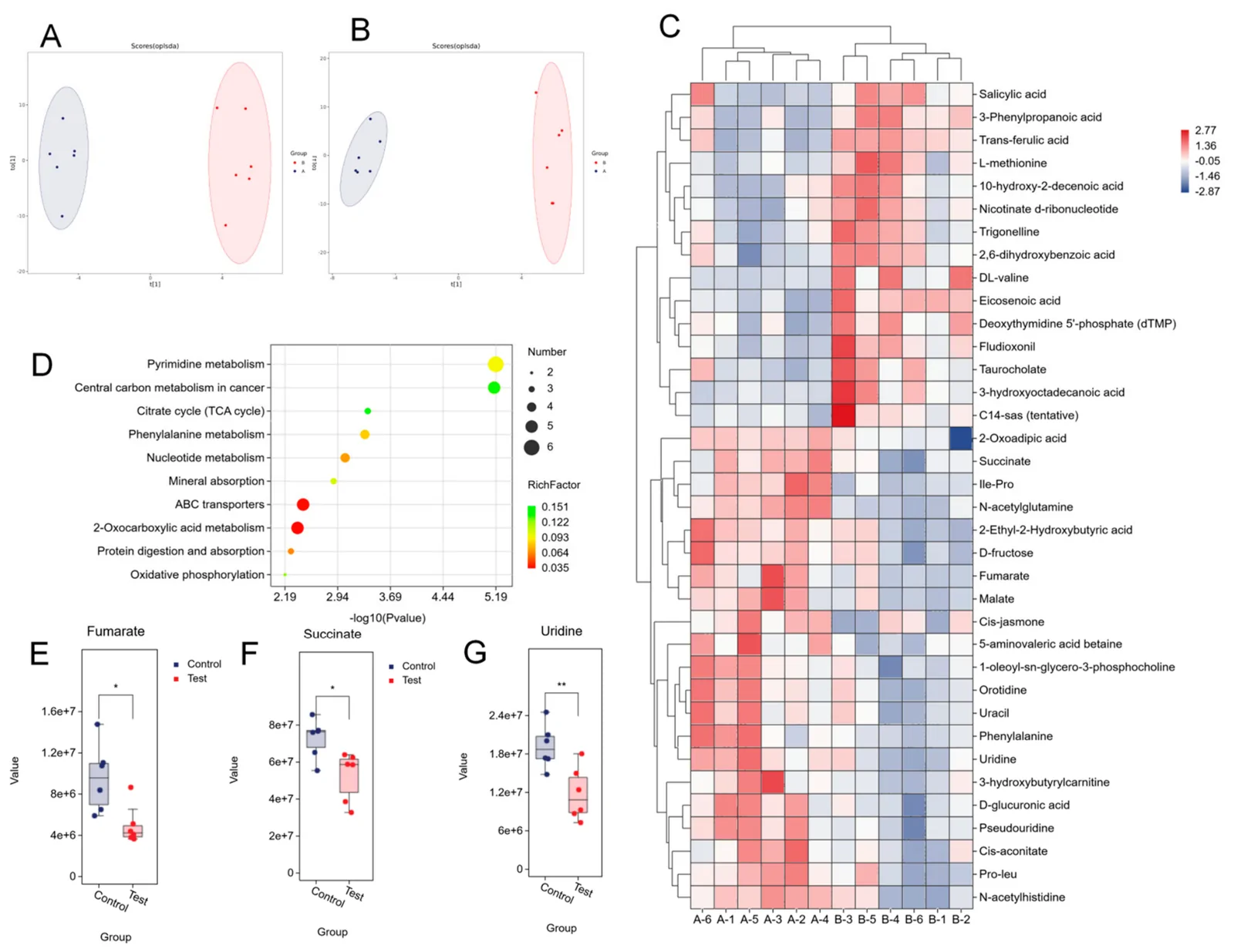
Nitrous oxide alters plasma metabolites in mice (*n* = 6). (**A**,**B**): OPLS-DA Score map of plasma ((**A**): positive ion mode; (**B**): negative ion mode), (**C**): Heat map of differential metabolites, (**D**): Differential metabolite pathways enrichment analysis bubble chart in plasma, (**E**): Boxplot of Fumarate expression (* *p* < 0.05; ANOVA and independent-samples Student’s *t*-test), (**F**): Boxplot of Succinate expression (* *p* < 0.05; ANOVA and independent-samples Student’s *t*-test), (**G**): Boxplot of Uracil expression (** *p* < 0.01; ANOVA and independent-samples Student’s *t*-test).

Although behavioral tests indicate that repeated nitrous oxide exposure causes cognitive impairment in mice, the underlying mechanisms remain unclear. Therefore, we further characterized the metabolite profiles of mice using LC-MS/MS to investigate potential causes. The model established using PCA on control and experimental group samples proved stable and reliable. As demonstrated by OPLS-DA, distinct metabolite profiles were observed between the control group and the experimental group (Figure 5A,B). Employing criteria of *p* < 0.05 and |log_2_FC| ≥ 0.585, we identified 36 differentially expressed metabolites. Notably, 15 metabolites were upregulated and 21 were downregulated in the experimental group compared to the control group (Figure 5C, Table S2). KEGG pathway analysis was performed to further investigate the predicted biological functions of these differential metabolites. They were significantly enriched in pathways related to pyrimidine metabolism, the tricarboxylic acid cycle, and other metabolic processes (Figure 5D). Notably, levels of uracil, fumarate, and succinate were significantly reduced in the experimental group (Figure 5E–G). These findings indicate that prolonged nitrous oxide exposure disrupts metabolic profiles in the mouse brain.

### 2.6. Verification of Key Mechanisms

**Figure 6:**
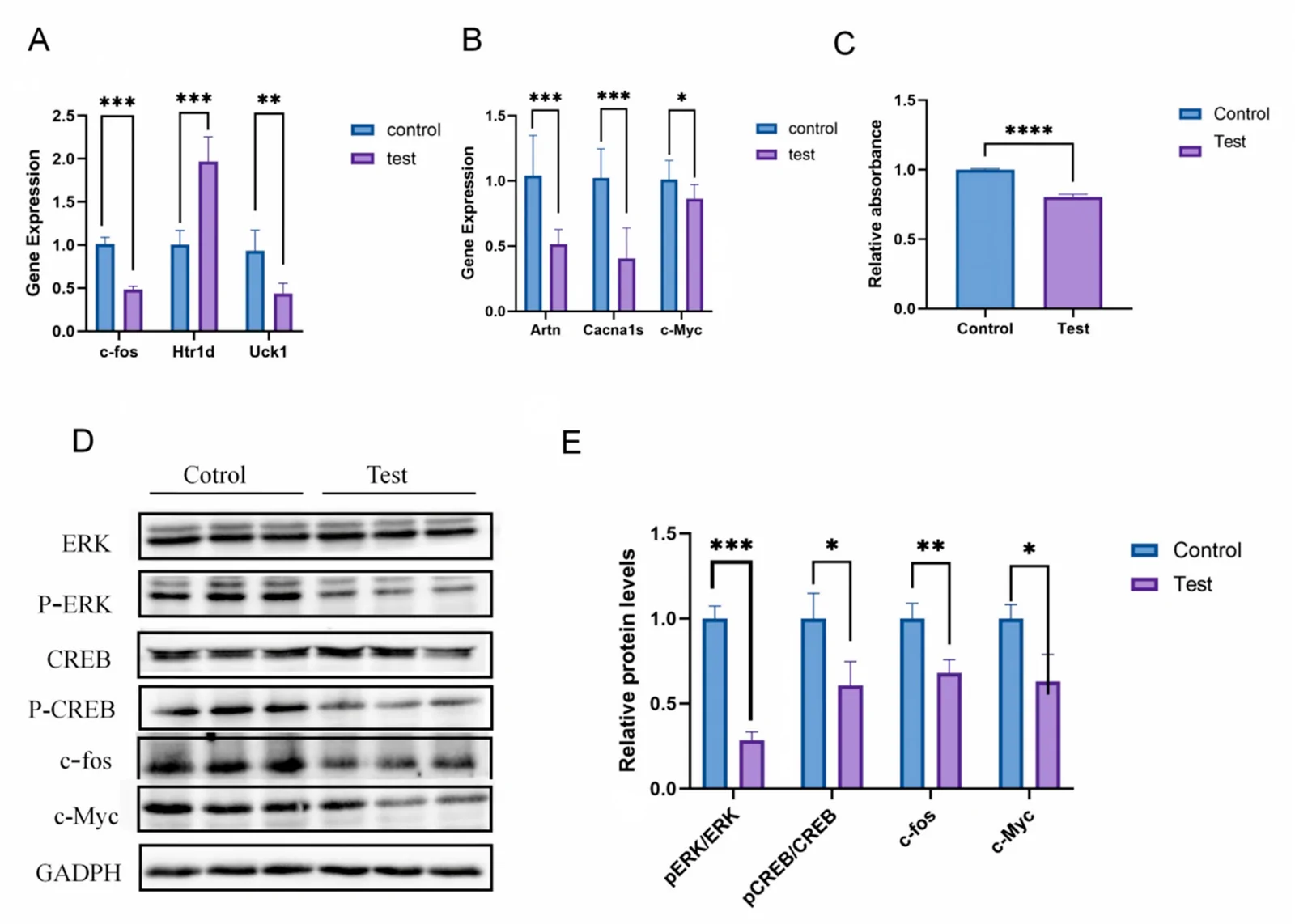
Nitrous oxide inhibits the expression of the MAPK pathway in the mice brain (*n* = 3). (**A**,**B**): Verification of representative hub genes by RT-qPCR. Data are shown as mean ± SEM. *** *p* < 0.005, ** *p* < 0.01, * *p* < 0.05; (ANOVA and independent-samples Student’s *t*-test) (**C**): ROS levels in the mouse hippocampus. Data are shown as mean ± SEM. **** *p* < 0.0001; (ANOVA and independent-samples Student’s *t*-test) (**D**,**E**): Protein expression of ERK, p-ERK, CREB, p-CREB, c-fos, and c-Myc in the mice hippocampus, *n* = 3 mice per group. Values are presented as mean ± SEM. * *p* < 0.05, ** *p* < 0.01, *** *p* < 0.001. (ANOVA and independent-samples Student’s *t*-test).

To better understand the molecular mechanisms underlying nitrous oxide-induced cognitive impairment, we performed real-time quantitative PCR analysis on hippocampal tissue to investigate the mRNA expression levels of key genes involved in the *MAPK* signaling pathway and pyrimidine metabolism pathway—specifically *c-fos*, *Cacna1s*, *Artn*, c*-myc, Htr1d*, and *Uck1*. Results showed that compared to the control group, *c-fos*, *Cacna1s*, *Artn*, *c-myc*, and *Uck1* were downregulated in the hippocampi of experimental mice, while Htr1d exhibited upregulation (Figure 6A,B). In addition, we measured ROS levels in the hippocampi of mice in the control and experimental groups and found that the levels in the experimental group were significantly lower than those in the control group (Figure 6C). Additionally, we examined the expression levels of significantly differentially expressed *MAPK* pathway-related proteins, including ERK, CREB, and their phosphorylated forms, along with their downstream targets c-fos and c-Myc. We found that p-ERK, p-CREB, c-fos, and c-Myc expression levels were reduced in the experimental group (Figure 6D,E). Inhibition of p-ERK/p-CREB signaling downregulates synaptic protein expression, potentially contributing to cognitive impairment.

## 3. Discussion

In recent years, adverse reactions caused by nitrous oxide inhalation have garnered increasing attention. However, research on nitrous oxide-induced cognitive impairment has primarily focused on clinical case analyses [16], with few systematic and comprehensive investigations into its key mechanisms and targets. Toxicological data remain insufficient, and the underlying mechanisms are still unclear. Recently, multi-omics technologies have enabled unbiased, high-throughput analysis of multiple targets simultaneously, offering new opportunities for drug safety assessment [17,18]. This study focuses on the effects of long-term nitrous oxide exposure on cognitive function in mice and its potential mechanisms. By integrating behavioral assessment, multi-omics analysis, and molecular validation, we address this critical knowledge gap to elucidate the mechanisms underlying the cognitive impairment caused by prolonged nitrous oxide exposure in mice. Our behavioral data indicate that prolonged nitrous oxide exposure impairs cognitive function in mice. Mice in the nitrous oxide-exposed group exhibited significantly reduced central zone activity and demonstrated impaired spatial memory in the novel object recognition test and water maze test. These behavioral phenotypes are consistent with studies reporting drug exposure-induced cognitive impairment. As a critical brain region for learning and memory, structural damage to the hippocampus is a common pathological feature of cognitive dysfunction [19]. Our findings reveal that prolonged nitrous oxide exposure induces significant neuropathological alterations in hippocampal tissue, primarily manifested as neuronal swelling and disorganized satellite cell arrangement—hallmarks of neurotoxic histology, these morphological changes may lead to abnormalities in information encoding and retrieval processes, thereby manifesting as cognitive dysfunction at the behavioral level [20]. Further analysis revealed significant downregulation (*p* < 0.05) of c-fos expression—an immediate early gene closely associated with synaptic plasticity and neuronal activity—in hippocampal tissue. This suggests nitrous oxide exposure may impair normal hippocampal neuronal function, thereby affecting synaptic plasticity. These findings confirm the neurotoxic effects of nitrous oxide on hippocampal structure and function, though the specific molecular mechanisms underlying nitrous oxide-induced cognitive impairment warrant further investigation.

At the molecular regulatory mechanism level, transcriptomic sequencing results revealed that prolonged nitrous oxide exposure significantly alters the gene expression profiles of synaptic plasticity-related signaling pathways in mouse hippocampal tissue. Differentially expressed genes identified through KEGG pathway enrichment analysis were significantly enriched in serotonergic synaptic pathways, suggesting that nitrous oxide exposure may modulate neuronal synaptic plasticity through disruption of serotonergic neural signaling. To validate the reliability of transcriptomic analysis, we further employed real-time quantitative polymerase chain reaction (qPCR) to perform targeted validation of key regulatory genes within the serotonergic synaptic pathway. Results showed that mRNA expression of serotonin receptor 1D (HTR1d) was significantly upregulated in hippocampal tissue from nitrous oxide-exposed mice (*p* < 0.001), confirming the central role of this pathway in nitrous oxide neurotoxicity. As a key member of the serotonin (5-HT) receptor family, HTR1d is primarily localized to the presynaptic membrane of serotonergic neurons and other neuronal types, where it functions as a presynaptic autoreceptor [21]. Upon activation, HTR1d inhibits presynaptic 5-HT release through negative feedback regulation, leading to a significant reduction in effective 5-HT concentration in the synaptic cleft. The attenuation of serotonergic signaling not only disrupts the activation equilibrium of postsynaptic membrane receptors but also further impedes the normal renewal process of synaptic membranes and the transmission efficiency of lipid signaling molecules. Ultimately, this compromises the maintenance of neuronal homeostasis and the capacity for tissue repair, leading to diminished synaptic plasticity [22]. Consequently, this pathway emerges as one of the key molecular mechanisms underlying nitrous oxide-induced cognitive dysfunction.

Additionally, alterations in brain metabolome profiles highlight three metabolites—fumarate, uracil, and succinate—that may contribute to cognitive decline induced by long-term nitrous oxide exposure. Integrated transcriptomics and metabolomics analyses suggest that the TCA cycle, MAPK signaling pathway, and pyrimidine metabolism may interact to precipitate cognitive impairment. Our findings provide research evidence supporting the notion that nitrous oxide intake may lead to cognitive impairment.

The TCA cycle serves as the central hub for energy metabolism and metabolic transformation in living organisms. The brain relies on glucose catabolism to generate ATP for sufficient energy supply. As one of the primary pathways for ATP production following glucose breakdown in the brain, alterations in the TCA cycle significantly impact cerebral metabolic rates [23]. Metabolic intermediates of the TCA cycle also serve as key precursors for complex biosynthesis. In our study, levels of fumarate and succinate were significantly decreased (*p* < 0.05). Succinate is produced via the hydrolysis of succinate-CoA, representing the sole ATP-generating step within the TCA cycle. This finding indicates that prolonged nitrous oxide exposure disrupts energy metabolism in mice. Dysfunction in the TCA cycle alters the efficiency of the mitochondrial electron transport chain, leading to abnormal production of ROS. ROS act as activators of the MAPK pathway, and severe imbalance disrupts normal signaling within the MAPK pathway [24]. Our experiments have also confirmed this view.

Mitogen-activated protein kinase (MAPK) is a serine/threonine protein kinase comprising four parallel signaling pathways, among which the ERK1/2 pathway was the first to be identified and plays a crucial role in regulating cognitive impairment [25,26]. Upon activation, this pathway regulates gene expression related to synaptic plasticity and memory formation by phosphorylating the CREB transcription factor. Abnormal CREB function—such as reduced activity or excessive activation—directly disrupts cognitive-related molecular and cellular mechanisms, leading to cognitive impairment [27,28,29]. Wu [30] observed that adolescent mice exposed to chronic lead exhibited behavioral changes indicative of cognitive impairment, accompanied by downregulated pCREB expression in the hippocampus. This suggests that prolonged lead exposure suppresses pCREB activation, thereby causing cognitive dysfunction. Furthermore, MAPK/ERK pathway function is regulated by multiple upstream and parallel molecules. This study observed downregulation of Artn, a glial cell-derived neurotrophic factor family ligand, and Cacna1s, the gene encoding the L-type voltage-gated calcium channel α1s subunit. Artn plays a crucial role in neurodevelopment, neuronal survival, and synaptic function maintenance. Its reduced expression may further inhibit MAPK/ERK signaling, increasing neuronal vulnerability under stress conditions [31]. The protein encoded by Cacna1s constitutes a key component of calcium ion channels, being essential for synaptic plasticity, gene transcription, and regulation of neuronal excitability; Its reduced expression often indicates diminished calcium influx, and calcium signaling is a key initiator of the Ras-MAPK/ERK cascade [32]. These changes collectively indicate multi-level inhibition of the MAPK/ERK pathway. Huang [33] found significant suppression of the MAPK/ERK pathway in rats with anesthesia-induced cognitive impairment, consistent with our findings.

In this study, we observed a significant decrease in ROS levels in the hippocampus following repeated nitrous oxide exposure, a finding that deviates from the classical pattern of elevated ROS commonly reported in most neurotoxicity models [34,35]. We propose that the 28-day exposure period may have triggered an adaptive compensatory response, characterized by upregulation of endogenous antioxidant defense systems via the Nrf2/ARE pathway and downstream enzymes including SOD, CAT, and GPx. If the enhanced scavenging capacity exceeds the rate of ROS generation, the net steady-state ROS level could decrease [36]. Furthermore, consistent with our metabolomic data, the significant downregulation of TCA cycle intermediates—succinate and fumarate—suggests impaired mitochondrial electron transport chain (ETC) activity. Given that the ETC is the primary source of intracellular ROS, reduced ETC flux would predictably diminish electron leakage and consequently lower ROS production [37]. Thus, the observed decrease in ROS levels is mechanistically coherent with the mitochondrial energy metabolic disruption identified in our study.

In our study, c-Myc expression was decreased following inhibition of the MAPK pathway. c-Myc is a key proto-oncogene in the human genome, encoding a protein involved in regulating cell proliferation, differentiation, and apoptosis. It has been extensively studied in tumorigenesis [38,39]. Zhang [40] investigating the molecular mechanisms by which cadmium exposure affects cognition, found that cadmium disrupts mitochondrial homeostasis by inhibiting the Wnt3a/β-catenin/c-Myc signaling axis, leading to neural stem cell (NSC) aging and cognitive dysfunction. Activation of this pathway significantly alleviates cadmium’s neurotoxic effects, identifying it as a therapeutic target. β-catenin/c-Myc signaling axis to disrupt mitochondrial homeostasis, thereby inducing NSC senescence and cognitive impairment. Activation of this pathway significantly mitigates cadmium’s neurotoxic effects, offering novel insights for studying cadmium-mediated cognitive dysfunction. c-Myc directly activates transcription of key enzymes in the de novo pyrimidine synthesis pathway, such as CAD and Uck1. Reduced c-Myc expression decreases the activity of these regulated enzymes, thereby impairing overall de novo pyrimidine synthesis capacity. Uck1 catalyzes the first irreversible rate-limiting step in pyrimidine nucleoside salvage and is widely expressed in all tissues, with particularly elevated expression in the brain. When its expression is downregulated by c-Myc, endogenous UMP synthesis becomes insufficient, and exogenous uridine utilization is impaired, ultimately leading to depletion of intracellular uridine/UMP/CTP pools [41]. Pyrimidines are essential components of nucleic acid synthesis, and abnormalities in their metabolism are associated with neurological dysfunction and psychiatric disorders. The nucleotides produced from their metabolism (primarily derivatives of uracil and cytosine) serve as raw materials for DNA/RNA synthesis [42]. Reduced levels of these nucleotides decrease RNA synthesis, directly impacting the synthesis of new proteins within neurons and ultimately contributing to cognitive impairment [43].

It is well established that nitrous oxide exerts its neurotoxic effects primarily through the irreversible oxidation of the cobalt ion in vitamin B_12_ (cobalamin), thereby inactivating methionine synthase—a key enzyme in the methionine–homocysteine cycle. Inhibition of this enzyme leads to elevated homocysteine levels and reduced S-adenosylmethionine (SAM) availability, which in turn triggers oxidative stress [44], impairs myelin maintenance, and disrupts neuronal function. Our multi-omics findings—including disrupted TCA cycle intermediates (succinate and fumarate), downregulated MAPK/ERK/CREB signaling, and perturbed pyrimidine metabolism—may be interpreted as downstream consequences of vitamin B_12_/methionine synthase inhibition. SAM is critical for mitochondrial function and redox homeostasis; its depletion compromises electron transport chain activity and reduces TCA cycle flux, consistent with the decreased succinate and fumarate levels observed in our study [45]. Moreover, elevated homocysteine is known to induce oxidative stress and activate stress-responsive kinases, which may impair the ERK/CREB pathway and compromise synaptic plasticity—a notion supported by our observed reductions in p-ERK and p-CREB expression.

**Figure 7:**
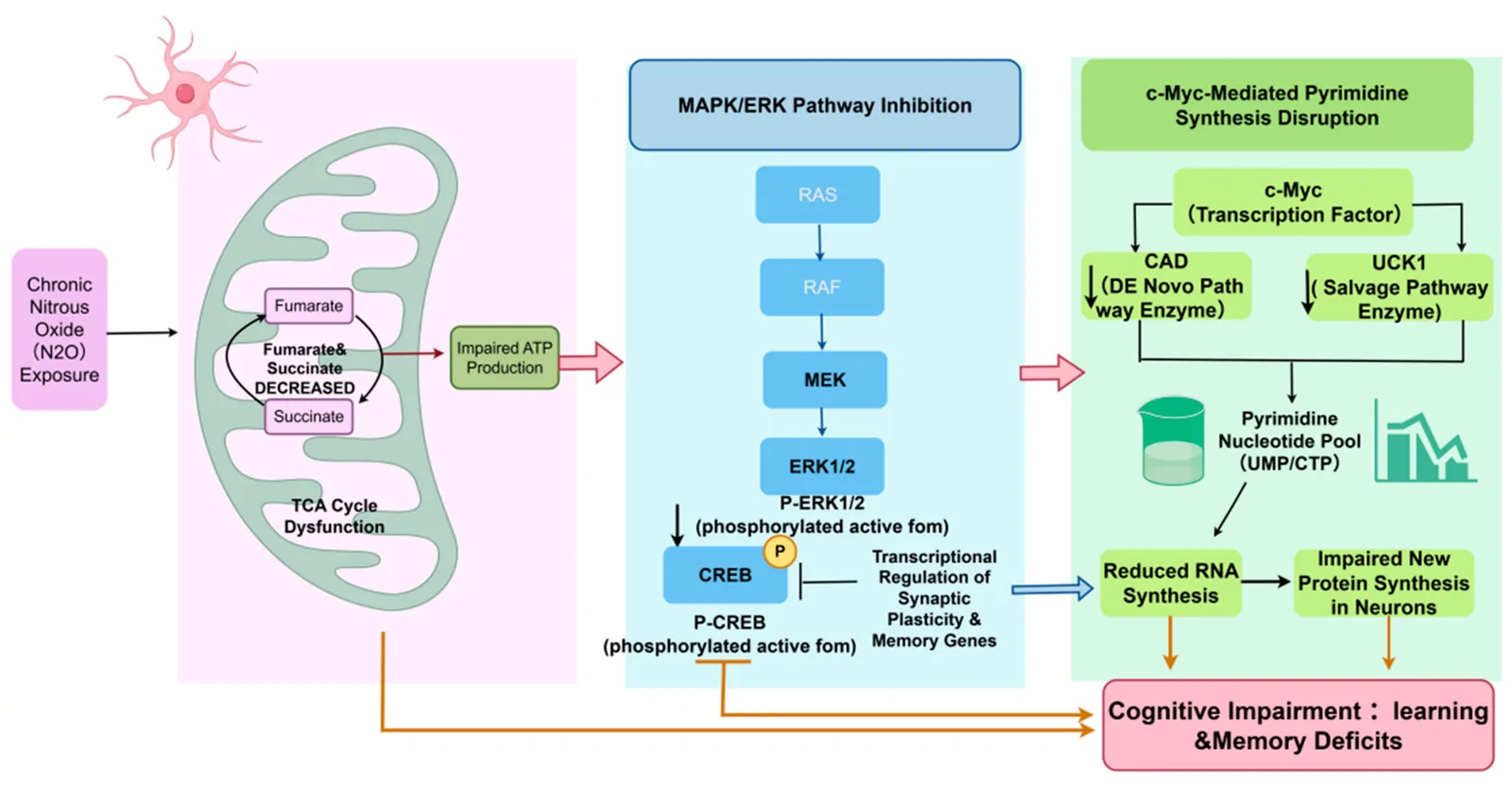
Mechanism diagram of nitrous oxide-induced cognitive impairment.

The results of this study indicate that the levels of succinate and fumarate in the serum of mice in the nitrous oxide experimental group were significantly downregulated. These changes directly interfere with the mitochondrial TCA cycle process, leading to mitochondrial energy metabolism disorders, and indirectly inhibit the MAPK neuroplasticity signaling pathway through a cascade reaction. Concurrently, the expression of c-Myc—a core regulator of uracil phosphoribosyltransferase (UCK1), the key rate-limiting enzyme in the pyrimidine salvage pathway—was significantly reduced. This decrease led to diminished uracil levels, subsequently inducing pyrimidine metabolism disorders. Abnormal pyrimidine metabolism further exacerbates mitochondrial energy metabolism imbalance. Multi-omics integration analysis further confirms that nitrous oxide can ultimately trigger cognitive dysfunction in experimental animals by targeting the regulation of the tricarboxylic acid (TCA) cycle and MAPK signaling pathway, mediating pyrimidine metabolic reprogramming, and ultimately causing energy metabolism network imbalance (Figure 7).

This study established a nitrous oxide-induced cognitive impairment model through behavioral testing and multi-omics integrative analysis. Transcriptome KEGG pathway enrichment analysis revealed that genes involved in cognitive impairment were significantly downregulated following nitrous oxide induction, with marked suppression of the MAPK pathway. Concurrently, compared to the control group, the experimental group exhibited downregulation in pyrimidine metabolism and the tricarboxylic acid cycle, particularly prominent in the metabolome, with c-Myc emerging as a potential target. Thus, our study lays a solid foundation for further investigating the specific role of nitrous oxide-induced cognitive impairment at the metabolic regulatory level. However, this study has limitations. This experiment primarily investigates the neurotoxicity of nitrous oxide in male mice and the resulting cognitive impairment; however, this study only employed a single time point and a single concentration level. This approach limits our ability to assess the dose–response relationship and temporal dynamics. In future studies, we will conduct experiments using female mice to elucidate the gender-specific effects of nitrous oxide on cognitive function in mice. Since the relationship between metabolites and their associated genes remains at a predictive stage, further validation through target activation experiments is warranted to provide new insights for the treatment of nitrous oxide-induced cognitive impairment.

## 4. Materials and Methods

### 4.1. Animals

Healthy 7-week-old male *C57BL/6* mice were purchased from Beijing Changyang Xishan Breeding Farm. Mice were maintained under a 12 h light/dark cycle with free access to standard laboratory chow and water for one week. Cages were cleaned daily throughout the experiment. All efforts were made to minimize animal suffering and to reduce the number of animals used. The N_2_O concentration of 90,000 ppm was selected based on a dose conversion extrapolated from a 60 kg adult human consuming 50 units of 8 g nitrous oxide cartridges per day, aiming to simulate chronic heavy recreational use while avoiding acute hypoxia [46]. All experimental procedures were reviewed and approved by the Ethical Committee for Use of Laboratory Animals of the Shanxi Medical University (SYDL2025013). All studies were carried out in accordance with Guidance on the operation of the Animals (Scientific Procedures) Act 1986 and associated guidelines.

### 4.2. Establishment of Cognitive Impairment Model

Mice were randomly divided into an air control group and a nitrous oxide experimental group (*n* = 9 per group). The experimental group received 90,000 ppm nitrous oxide (Henan Yuanzheng Specialty Gases Co., Xinxiang, China) for 28 days, administered twice daily for 1 h per session. The air control group underwent parallel exposure to air for 28 days, also twice daily for 1 h per session. Behavioral tests were conducted on days 22–28 to confirm the successful establishment of the cognitive impairment model.

### 4.3. Behavioral Experiment

#### 4.3.1. Open Field Test

The open field test was conducted using a 50 cm × 50 cm specialized open field apparatus (Shanghai Jiliang Software Technology Co., Ltd., Shanghai, China). At the start of the experiment, mice were gently placed near the wall. A specialized camera system recorded behavior data such as the mice’s activity paths and movement duration. The test duration was 600 s, during which the total distance traveled was automatically recorded.

#### 4.3.2. Novel Object Recognition Test

First, mice were placed in a 50 cm × 50 cm cube-shaped box (Shanghai Jiliang Software Technology Co., Ltd., Shanghai, China). Objects A and B, identical in appearance, were pre-positioned at opposite ends along the diagonal from the upper left to lower right corners. After 10 min of free exploration, the mice were removed and returned to their cage. Object B was then replaced with a new object C of different appearance. Following a 60 min rest period, the mice were reintroduced to the behavioral apparatus for another 10 min of free exploration, during which the session was videotaped. Post-experiment analysis calculated the duration and frequency of the mice’s exploration of the new and old objects.

#### 4.3.3. Morris Water Maze Test

All mice underwent a 5-day Morris water maze test (Shanghai Jiliang Software Technology Co., Ltd., Shanghai, China). The first 4 days primarily involved spatial navigation training, and the final day was dedicated to spatial exploration testing. During navigation training, a 5.0 cm × 5.0 cm platform was placed approximately 1 cm below the surface in the water maze (a circular pool with a diameter of 90 cm). Titanium dioxide was added to the water and stirred until uniformly dispersed, to create an opaque solution. Mice were randomly placed facing the wall in one of four quadrants. If the time from placement to successful platform access exceeded 60 s, the mice were manually guided to the platform. After reaching the platform, the mice were reinforced by being remain there for 10 s to consolidate memory, then dried and cared for. Each mouse underwent four training sessions daily. A software system recorded each mouse’s swimming trajectory. On Day 5, the spatial exploration test was initiated. The platform was removed, and the quadrant opposite the platform was designated as the entry point. The mice were allowed to swim freely and explore for 60 s. Finally, the time spent by each mouse in the target quadrant was analyzed and counted.

All behavioral experiments were conducted by the same researcher at night, under a quiet experimental environment.

### 4.4. Hematoxylin and Eosin Staining

Paraffin-embedded brain tissue sections were processed as follows: dewaxed with xylene (2 × 10 min), hydrated with graded ethanol (100% → 70%), and rinsed with distilled water. Subsequently, sections were stained with hematoxylin (8–15 min), differentiated in acid-ethanol (30 s), washed in water (30 min), and counterstained with eosin (5 min). Subsequently, sections were dehydrated through graded ethanol (70% → 100%), cleared with xylene, mounted with neutral balsam, and photographed under a microscope.

### 4.5. Immunofluorescence Staining (IF)

Brain tissues were fixed, sectioned, and deparaffinized to water, followed by antigen retrieval with ethylenediaminetetraacetic acid (EDTA) buffer. Then 10% serum was added dropwise, and sections were blocked at 37 °C for 30 min, followed by incubation with primary antibody NeuN (1:100, PTG) diluted in TBST at 4 °C overnight. Sections were then incubated with secondary antibody Alexa Fluor 488 goat anti-rabbit IgG (H + L) (1:400, Thermo Fisher Scientific, Waltham, MA, USA) for 45 min at 37 °C. Nuclei were stained with DAPI working solution for 5 min in the dark, after which sections were coverslipped, observed under a light microscopy, and imaged. Fluorescence images were acquired using a Nikon Eclipse microscope (Nikon Corporation, Tokyo, Japan) slide scanner and analyzed with ImageJ software (Version 1.53, National Institutes of Health, Bethesda, MD, USA).

### 4.6. Metabolomics Analysis

A 100 μL of whole blood sample was transferred to a 1.5 mL microcentrifuge tube. Homogenization beads and 500 μL extraction solution (methanol: acetonitrile: water = 2:2:1 (*v*/*v*)) were added, then 1 mL methanol/acetonitrile/water solution (2:2:1, *v*:*v*:*v*) were used to precipitate proteins at 4 °C. After vortex mixing, sonicated the sample mixture for 30 min (maintain low temperature using ice). The sonicated mixture was allowed to stand at 20 °C for 10 min, then centrifuged at 14,000× *g* for 20 min at 4 °C. The supernatant was collected and dried under vacuum. For instrumental analysis, the dried residue was reconstituted in 100 µL of aqueous acetonitrile solution. After vortex mixing, the sample was centrifuge at 14,000× *g* for 15 min at 4 °C, and filtered through a 0.22 μm organic-phase membrane. The prepared sample was then subjected to Agilent 1260(Agilent Technologies, Santa Clara, CA, USA)—AB SCIEX Triple TOF6600(AB SCIEX, Framingham, MA, USA) HPLC-MS/MS analysis To ensure instrument stability and data reliability, quality control samples were randomly prepared and analyzed throughout the process.

### 4.7. Transcriptomic Analysis

Total RNA was extracted from hippocampal tissue using TRIzol^®^ reagent (Invitrogen, Thermo Fisher Scientific, Carlsbad, CA, USA). PE libraries were prepared according to the ABclonal mRNA-seq Lib Prep Kit protocol. mRNA was purified from 1 μg total RNA using oligo magnetic beads, followed by mRNA fragmentation in ABclonal First Strand Synthesis Reaction Buffer. Subsequently, using mRNA fragments as templates, the first strand of cDNA was synthesized with random primers and reverse transcriptase (RNase H). The second strand of cDNA was synthesized using DNA polymerase I, RNase H, buffer, and dNTPs. The synthesized double-stranded cDNA fragments were ligated with adapter sequences for PCR amplification. The PCR products were purified, and library quality was assessed using an Agilent Bioanalyzer 4150 TapeStation system (Agilent Technologies Inc., Santa Clara, MA, USA). Finally, sequencing was performed on an MiSeq-T7 platform (Illumina, Inc., San Diego, CA, USA).

### 4.8. Western Blotting Analysis

Hippocampal tissue was cryogenically ground in liquid nitrogen and added to a mixture of protease and phosphatase inhibitors and 1 × RIPA buffer. Protein concentration was determined using the BCA Protein Assay Kit. Proteins were separated on a 10% gel and subsequently transferred to a polyvinylidene fluoride membrane. The membrane was incubated with 5% BSA at room temperature for 1 h, followed by overnight incubation with the primary antibody at 4 °C on a shaking incubator. The membrane was washed three times for 10 min each in TBST (pH 7.5) containing 1% Tween 20, then incubated with the secondary antibody at room temperature for 1 h. The membrane was incubated with HRP-conjugated secondary antibody for 120 min. Proteins were visualized using ECL and analyzed with Image Lab^®^ (Bio-Rad Laboratories, Inc., Hercules, CA, USA).

### 4.9. RNA Extraction and Quantitative Real-Time PCR (RT-qPCR)

Total RNA from hippocampal tissue was extracted using the Tiangen RNA Easy Fast Animal Tissue Total RNA Extraction Kit (Tiangen, Beijing, China), followed by reverse transcription and quantification with the Super Plus Two-Step Reverse Transcription Premix Kit (Polymerase Bio M5, Beijing, China). PCR parameters were as follows: 10 min at 95 °C, followed by 40 cycles of 10 s at 95 °C and 20 s at 60 °C. Primer sequences are provided in Table S3. PCR was performed in triplicate technical replicates. Relative mRNA quantification was determined using the 2^−ΔΔCt^ method.

### 4.10. Data Analysis

Statistical analyses were performed using GraphPad Prism software (Version 8.0.2; GraphPad Software, Inc., La Jolla, CA, USA). The normality of data distribution in each group was assessed using the Shapiro–Wilk test. The homogeneity of variances between groups was evaluated using Brown-Forsythe test. Since both the normality and variance homogeneity assumptions were satisfied, independent-samples Student’s *t*-tests were employed to assess intergroup differences. A significance threshold of *p* < 0.05 was established, with values below this threshold considered statistically significant. All data are presented as mean ± standard deviation (SD). To ensure methodological reliability, all experiments were independently replicated three times.

## Data Availability

The data that support the findings of this study are included in the main article and its Supplementary Materials. Additional data underlying this study are available from the corresponding author upon reasonable request.

## Supplementary Materials

The following supporting information can be downloaded at: https://www.mdpi.com/article/10.3390/molecules31162844/s1, Figure S1: Westen Blot; Table S1: Different Genes; Table S2: Different metabolites; Table S3: Primer sequence.
