# Supplementary material for: Nitrous Oxide as a Recreational Drug of Abuse: Multi-Omics Insights into MAPK/ERK/CREB-Mediated Neurotoxicity and Energy Metabolism Collapse
Source: Molecules. 2026 Aug 14;31(16):2844. doi: 10.3390/molecules31162844 (PMC13515470; doi:10.3390/molecules31162844)
Supplement: Supplementary file 1 [file molecules-31-02844-s001.zip › Table S3. Primer Sequence.pdf]

Table S3 Primer Sequence

Name	5'to3'
c-myc-F	AGTGCTGCATGAGGAGACAC
c-myc-R	GCCTCTTCTCCACAGACACC
nppa-F	ATCCTGTGTACAGTGCGGTG
nppa-R	TACCGGCATCTTCTCCCTCCA
Htr1d-F	TTCACCACGGCACAGCTTAT
Htr1d-R	GTGTGTGTGGCTCTCATGGA
c-fos-F	TACTACCATTCCCCAGCCGA
c-fos-R	GCTGTCACCGTGGGGATAAA
ARTN-F	ATCTCAGTCTGGCCAGCCTA
ARTN-R	GAAGGAGACGGCCCTCATAGC
cPLA2-F	AACCCCGTGTGGAA TGAGAC
cPLA2-R	ACAGGGAATGTAGCTGTGCC
Uck1-F	CACCGCCTTTGTGAAACCAG
Uck1-R	TCCTGGCTTGGTCTGCCATA
Cacna1s-F	CAGGTAACAGGGGCAAAAGC
Cacna1s-R	TGGATCAGCATGGCAGTAGC
β-actin F	AGTGTGACGTTGACATCCGT
β-actin R	AGCTCAGTAACAGTCCGCCTA
