# Supplementary material for: Nitrous Oxide as a Recreational Drug of Abuse: Multi-Omics Insights into MAPK/ERK/CREB-Mediated Neurotoxicity and Energy Metabolism Collapse
Source: Molecules. 2026 Aug 14;31(16):2844. doi: 10.3390/molecules31162844 (PMC13515470; doi:10.3390/molecules31162844)
Supplement: Supplementary file 1 [file molecules-31-02844-s001.zip › Figure S1. westen blot.pdf]

C-fos

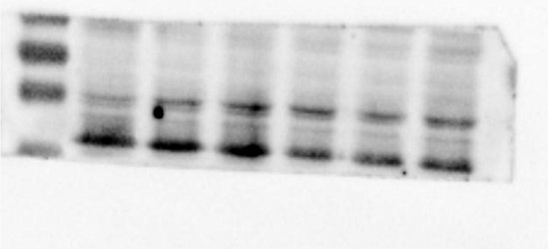

GADPH

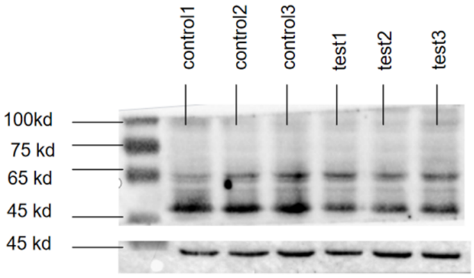

C-myc

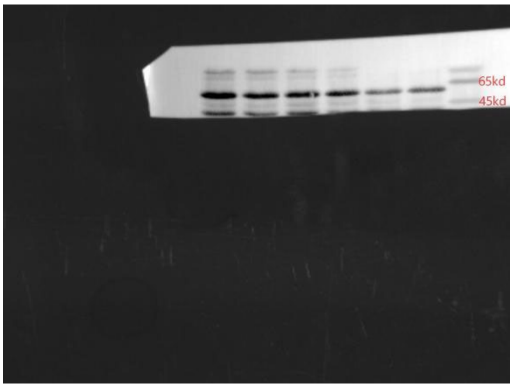

GADPH

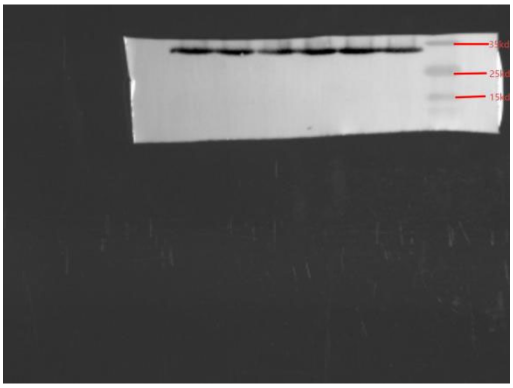

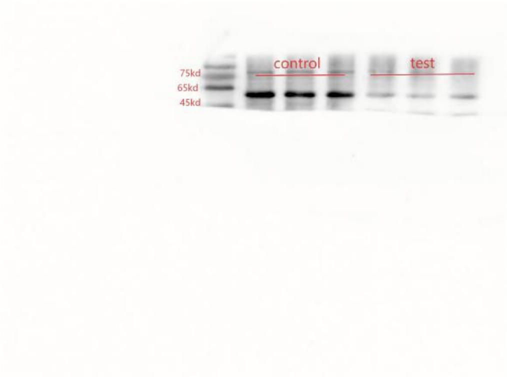

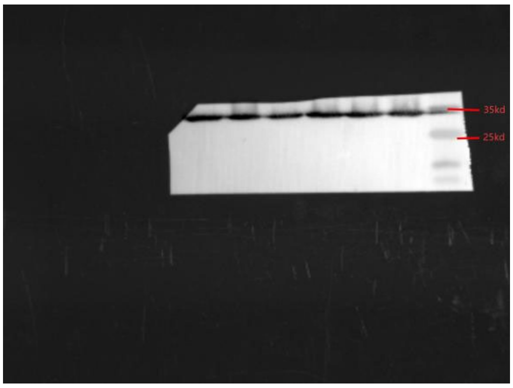

CREB

GADPH

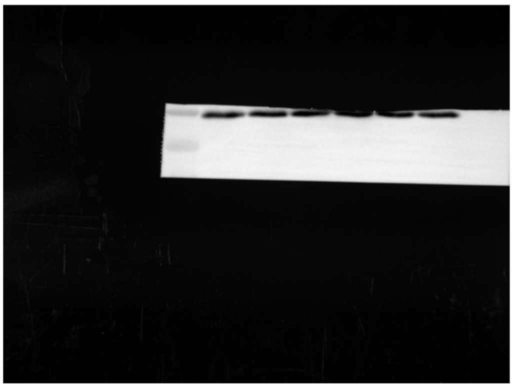

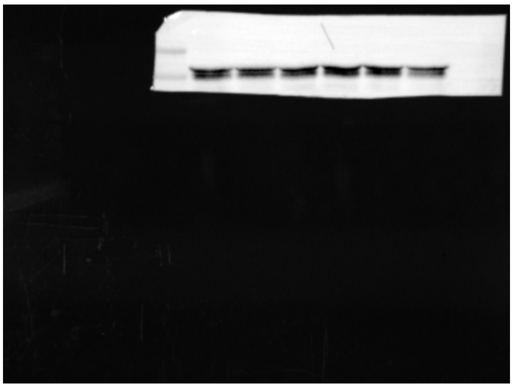

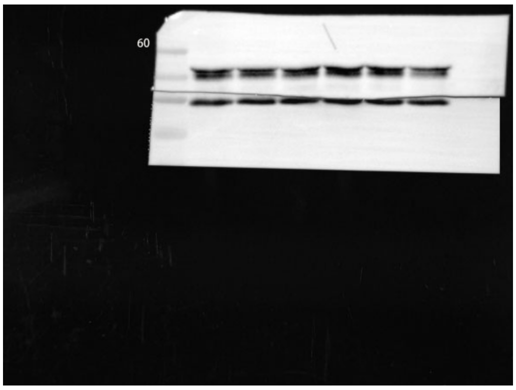
60

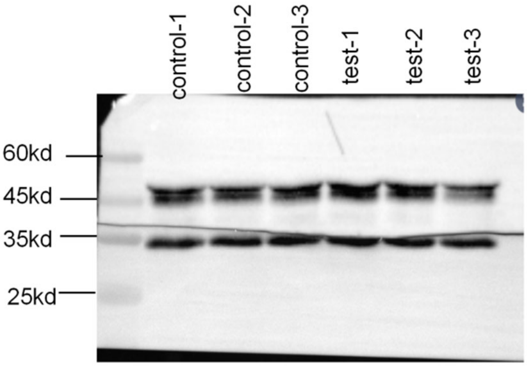
control-1
control-2
control-3
test-1
test-2
test-3
60kd
45kd
35kd
25kd

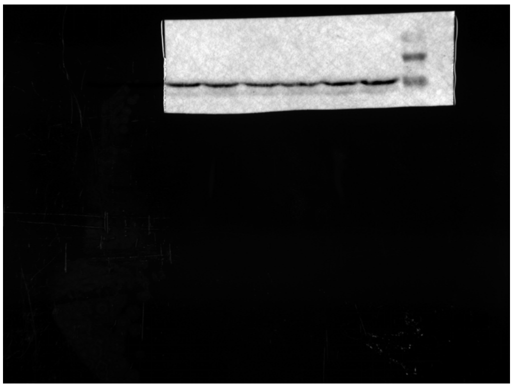

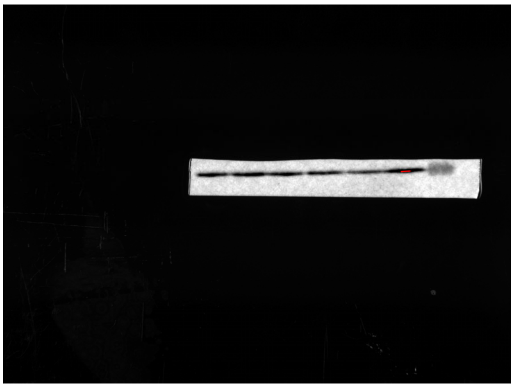

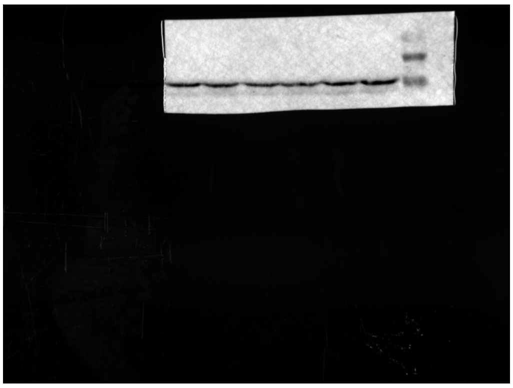

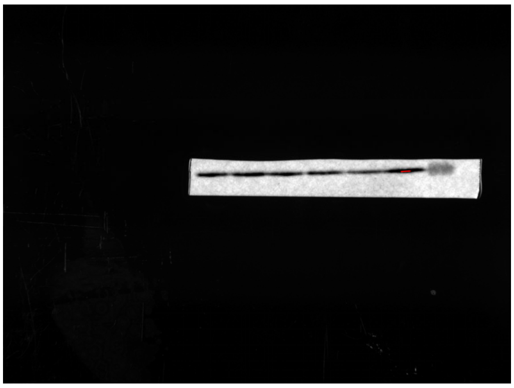

P-CREB GADPH

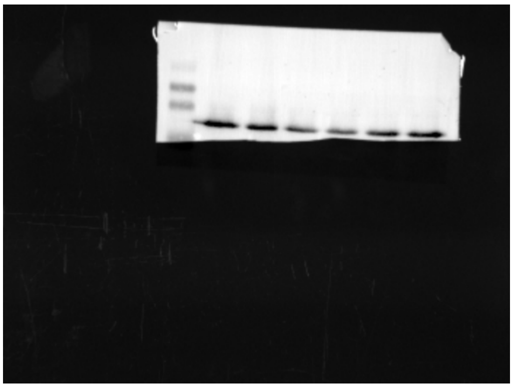

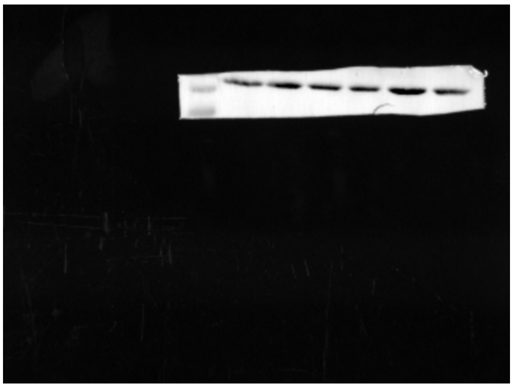

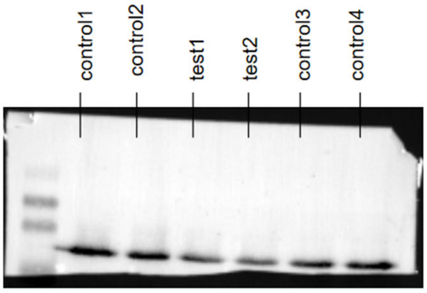
control1
control2
test1
test2
control3
control4

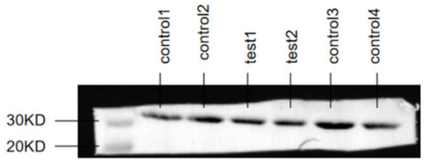
control1
control2
test1
test2
control3
control4
30KD
20KD

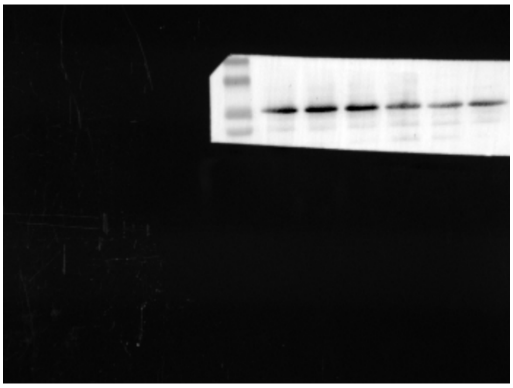

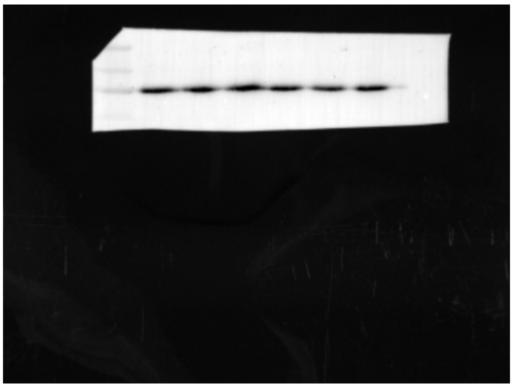

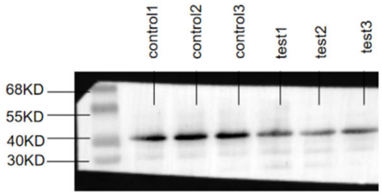
control1
control2
control3
test1
test2
test3
68KD
55KD
40KD
30KD

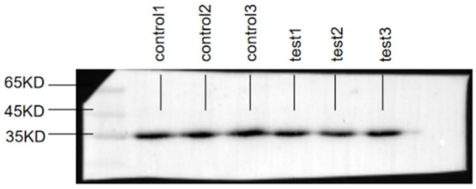
control1
control2
control3
test1
test2
test3
65KD
45KD
35KD

ERK

GADPH

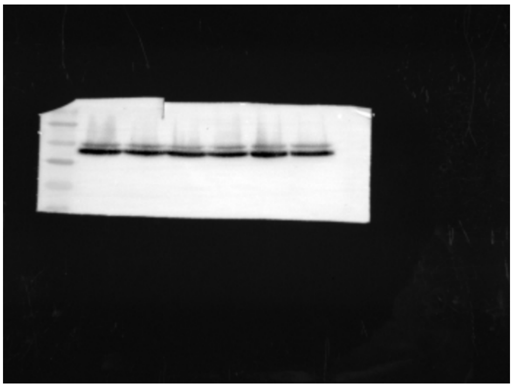

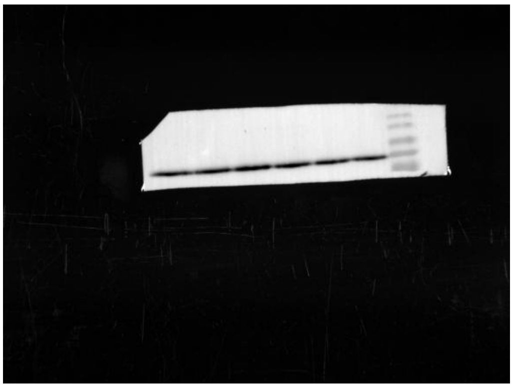

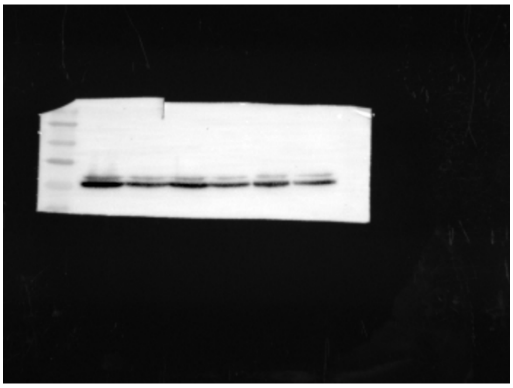

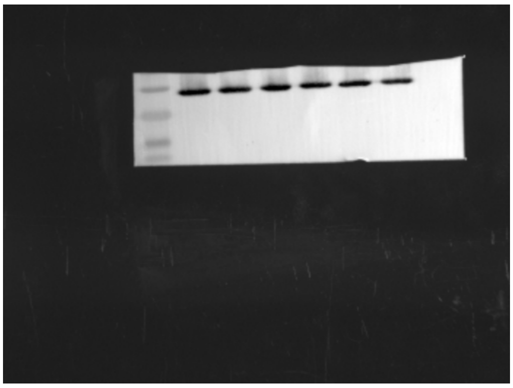

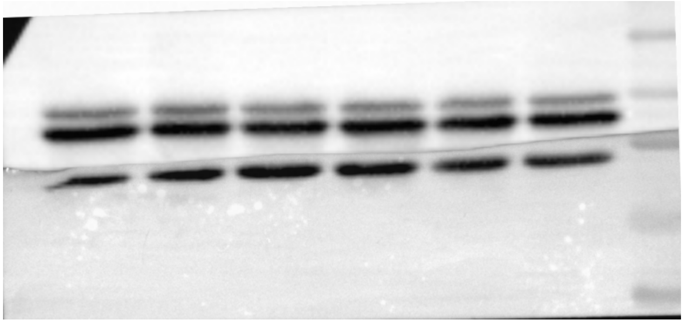

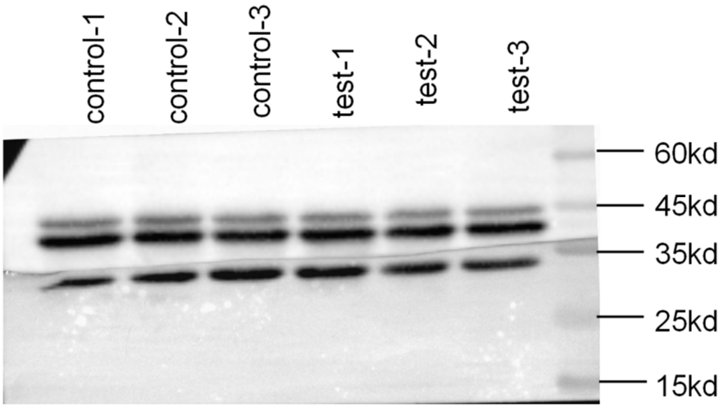
control-1
control-2
control-3
test-1
test-2
test-3
60kd
45kd
35kd
25kd
15kd

p-ERK

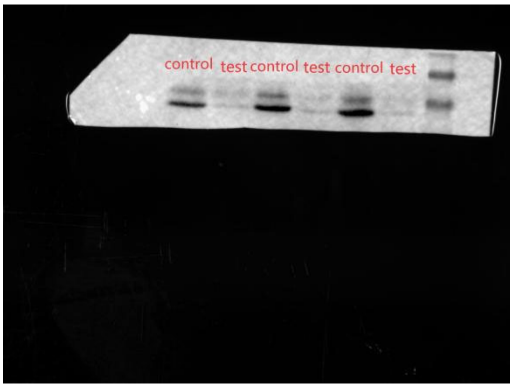
control test control test control test

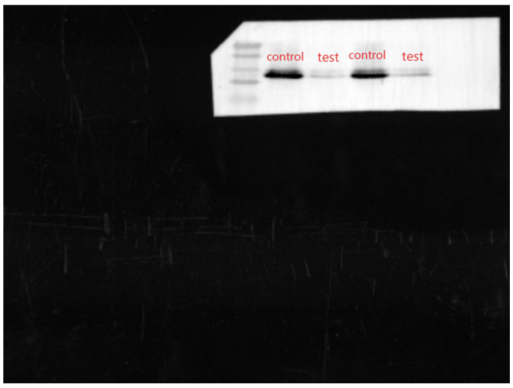
control test control test

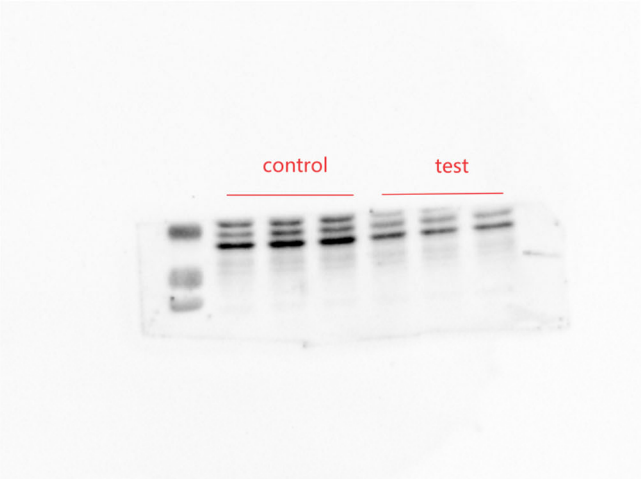
control test

GADPH

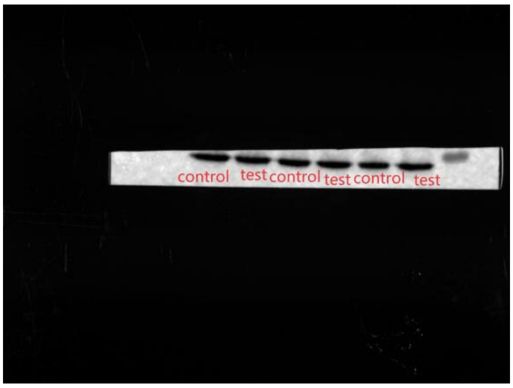
control test control test control test

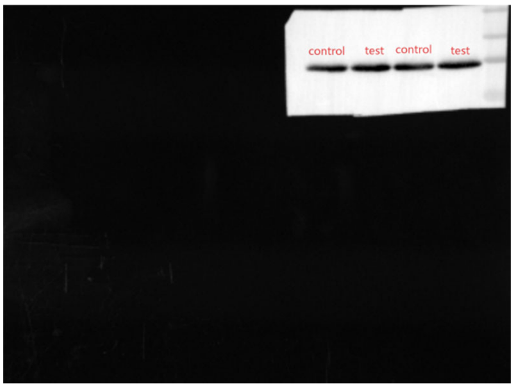
control test control test

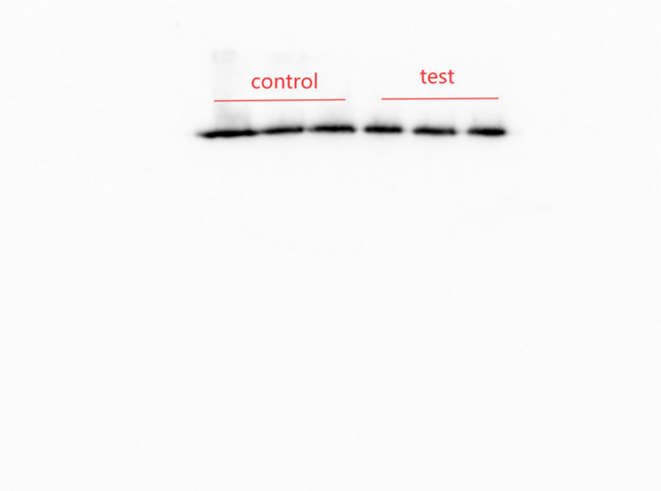
control test
